# Peer Review of “Impact of the COVID-19 Pandemic on the Mental Health of College Students in India: Cross-sectional Web-Based Study”

**DOI:** 10.2196/32953

**Published:** 2021-09-02

**Affiliations:** 1 NA NA, ON Canada

*This is a peer-review report submitted for the paper “Impact of the COVID-19 Pandemic on the Mental Health of College Students in India: Cross-Sectional Web-Based Study”.*

## Round 1 Review

### General comments

This paper [1] examined how the restrictions caused by COVID-19 impact students' mental health. A total of 324 students completed an online survey to report their fear of COVID-19 and other relevant mental health status. The findings indicate that more than half of the participants had strong fear of COVID-19 and that the fear of COVID-19 was associated with psychological distress of anxiety and depression. In general, the sample size was adequate and the statistical analyses are straightforward. However, the novelty of the present study is not clearly presented.

### Specific comments

#### Major comments

1. As I mentioned in the general comments, the novelty of the present study is not clearly presented. Specifically, ample evidence shows that fear of COVID-19 is associated with psychological distress, and such evidence includes samples from university students. Indeed, Pakpour and his colleagues have done a lot on this topic. Therefore, the authors should justify why there is a need to add their findings to the present literature.
2. There are many grammatical errors in the manuscript, such as “COVID-19 pandemic have created” and “This panic have led to the strong mental impact on them”. The authors should have a native English speaker carefully edit the submission to ensure the presentation quality.
3. The authors list “Brief Health Questionnaire” as one of the keywords; however, the present study does not use the Brief Health Questionnaire.
4. Until the *Methods* section, one can identify that the present study focuses on Indians. However, this information is given too late. In addition, the authors should provide some relevant information about COVID-19 in India during the data collection period.
5. The literature review of the present study is thin. As I mentioned earlier, there is ample evidence of the impacts of COVID-19 on mental health. However, the authors did not take references from the current evidence.
6. Additionally, many studies have reported the psychometric properties of the Fear of COVID-19 Scale (FCV-19S). If the authors want to report the concurrent validation of the FCV-19S, they should compare their findings with prior evidence on the psychometric properties of the FCV-19S.

#### Minor comments

1. Some tables use abbreviations, and the authors should spell out these abbreviations in a footnote.
2. Table 4 should have the correlation values in addition to the *P* values.
3. *P* values should never be 0.000; if the *P* values are really small, use *P*<0.001.
4. I cannot understand what the differences are between Table 4 and Table 5. Moreover, both tables are hard to understand.
5. Some references are not properly listed (eg, M.G H, Sonar NS, Ray B.)

## Round 2 Review

### General comments

This paper has improved according to the reviewers’ and editor's comments. In general, I think that the present form has some merits and is publishable. Although there are no major concerns in the revised version, several minor issues should be addressed in another round of revision.

### Specific comments

#### Minor comments

1. In the Abstract, I think that using * and *** to indicate significance levels is unnecessary because the authors have already provided the actual *P* values.
2. The authors should properly indicate that GAD-7 is the Generalized Anxiety Disorder Scale and PHQ-9 is the Brief Patient Health Questionnaire. The authors did not mention that GAD-7 is the Generalized Anxiety Disorder Scale and PHQ-9 is the Brief Patient Health Questionnaire in the main text. They only indicated this in the footnotes of the tables. Moreover, the authors sometimes used different terms to indicate the two scales (eg, in the Abstract, the authors mention Generalized Anxiety Scale instead of Generalized Anxiety Disorder Scale; sometimes the authors used Brief Patient Health Depression Questionnaire and sometimes Brief Patient Health Questionnaire). This is confusing.
3. The authors should properly indicate that GAD-7 is the Generalized Anxiety Disorder Scale and PHQ-9 is the Brief Patient Health Questionnaire. The authors did not mention that GAD-7 is the Generalized Anxiety Disorder Scale and PHQ-9 is the Brief Patient Health Questionnaire in the main text. They only indicated this in the footnotes of the tables. Moreover, the authors sometimes used different terms to indicate the two scales (eg, in the Abstract, the authors mention Generalized Anxiety Scale instead of Generalized Anxiety Disorder Scale; sometimes the authors used Brief Patient Health Depression Questionnaire and sometimes Brief Patient Health Questionnaire). This is confusing.
4. Reference: Pramukti I, Strong C, Sitthimongkol Y, Setiawan A, Pandin MGR, Yen C, Lin C, Griffiths MD, Ko N. Anxiety and suicidal thoughts during the COVID-19 pandemic: cross-country comparative study among Indonesian, Taiwanese, and Thai university students. *J Med Internet Res* 2020;22(12):e24487.
  In Table 4, please indicate the reference groups for the categorical independent variables.

## Abbreviations

FCV-19S: Fear of COVID-19 Scale
